# Improved reproducibility for myocardial ASL: Impact of physiological and acquisition parameters

**DOI:** 10.1002/mrm.29834

**Published:** 2023-09-05

**Authors:** Maša Božić‐Iven, Stanislas Rapacchi, Qian Tao, Iain Pierce, George Thornton, Christian Nitsche, Thomas A. Treibel, Lothar R. Schad, Sebastian Weingärtner

**Affiliations:** ^1^ Medical Faculty Mannheim Heidelberg University Mannheim Germany; ^2^ Department of Imaging Physics Delft University of Technology Delft The Netherlands; ^3^ CNRS, CRMBM Aix‐Marseille Université Marseille France; ^4^ Barts Heart Centre St Bartholomew's Hospital London UK; ^5^ Institute of Cardiovascular Science University College London London UK; ^6^ Division of Cardiology Medical University of Vienna Vienna Austria

**Keywords:** cardiac arterial spin labeling, cardiac magnetic resonance imaging, flow‐sensitive alternating inversion recovery, myocardial blood flow

## Abstract

**Purpose:**

To investigate and mitigate the influence of physiological and acquisition‐related parameters on myocardial blood flow (MBF) measurements obtained with myocardial Arterial Spin Labeling (myoASL).

**Methods:**

A Flow‐sensitive Alternating Inversion Recovery (FAIR) myoASL sequence with bSSFP and spoiled GRE (spGRE) readout is investigated for MBF quantification. Bloch‐equation simulations and phantom experiments were performed to evaluate how variations in acquisition flip angle (FA), acquisition matrix size (AMS), heart rate (HR) and blood $T_{1}$ relaxation time ($T_{1,B}$) affect quantification of myoASL‐MBF. In vivo myoASL‐images were acquired in nine healthy subjects. A corrected MBF quantification approach was proposed based on subject‐specific $T_{1,B}$ values and, for spGRE imaging, subtracting an additional saturation‐prepared baseline from the original baseline signal.

**Results:**

Simulated and phantom experiments showed a strong dependence on AMS and FA ($R^{2}$>0.73), which was eliminated in simulations and alleviated in phantom experiments using the proposed saturation‐baseline correction in spGRE. Only a very mild HR dependence ($R^{2}$>0.59) was observed which was reduced when calculating MBF with individual $T_{1,B}$. For corrected spGRE, in vivo mean global spGRE‐MBF ranged from 0.54 to 2.59 mL/g/min and was in agreement with previously reported values. Compared to uncorrected spGRE, the intra‐subject variability within a measurement (0.60 mL/g/min), between measurements (0.45 mL/g/min), as well as the inter‐subject variability (1.29 mL/g/min) were improved by up to 40% and were comparable with conventional bSSFP.

**Conclusion:**

Our results show that physiological and acquisition‐related factors can lead to spurious changes in myoASL‐MBF if not accounted for. Using individual $T_{1,B}$ and a saturation‐baseline can reduce these variations in spGRE and improve reproducibility of FAIR‐myoASL against acquisition parameters.

## 1 INTRODUCTION

First‐pass myocardial perfusion with cardiac MR (CMR) is widely used as the clinical gold standard for noninvasive assessment of myocardial ischemia.^1^, ^2^, ^3^, ^4^ However, the need for exogenous, gadolinium‐based contrast agents, limits the clinical applicability of first‐pass perfusion MRI. Since gadolinium is cleared from the body almost exclusively through the kidneys,^5^, ^6^ gadolinium‐based contrast agents are contraindicated in patients with renal dysfunction.^6^ Additionally, concerns have been raised about gadolinium accumulation in the brain following the repeated use of gadolinium‐based contrast agents, even in combination with healthy renal clearance.^7^, ^8^

Arterial Spin Labeling (ASL) offers a contrast‐agent free alternative for perfusion measurements, using magnetically labeled blood as an endogenous contrast.^9^, ^10^ ASL has been well established in neuro‐vascular applications and has steadily gained importance in quantifying cerebral blood flow over the last decades.^11^, ^12^ In cardiac applications, promising results have been achieved with myocardial ASL (myoASL): Reported myoASL‐based myocardial blood flow (MBF) values were in agreement with reference values from positron emission tomography (PET) gold standard measurements.^13^ Moreover, myoASL has shown to be sensitive to perfusion changes induced by either vasodilatory stress or when comparing normal and ischemic myocardial segments.^14^ However, due to a low signal‐to‐noise ratio, insufficient reproducibility and robustness have hampered more wide‐spread clinical translation of myoASL thus far.^15^

Typically, multiple pairs of tag and control images are acquired in an ASL measurement. In tag images magnetically labeled blood is flowing into the imaging volume, while no labeling is applied for control images. Subtracting tag from control yields perfusion weighted images, which can then be used to quantify the MBF.^13^, ^16^, ^17^ With signal differences between tag and control images of 1%–8%,^18^ myoASL is rendered very sensitive to physiological signal variations, such as those caused by cardiac or respiratory motion. This physiological noise (PN) was found to be the dominant noise source in myoASL.^13^ However, its ratio to thermal noise is highly dependent on the choice of imaging readout and acquisition parameters.^19^ In myoASL, the perfusion weighted signal is most commonly acquired using snapshot image readouts, where all k‐space lines are acquired in a single heartbeat. To obtain quantitative MBF, however, the perfusion weighted signal is modeled only based on the effects of the labeling preparation.^16^ As the imaging pulses perturb the magnetization signal, the image contrast can still be dependent on parameters related to image readout. This can cause a number of factors, including sequence parameters such as the acquisition flip angle or physiological parameters such as the heart rate variability, to affect the precision and bias of ASL measurements.

The objective of this study is to investigate the effect of physiological and acquisition‐related parameters on the bias and precision of quantitative myoASL measurements. Simulation and phantom experiments are used to evaluate the relative contribution of various confounders in balanced steady‐state free precession (bSSFP) and spoiled gradient‐echo (spGRE) based myoASL. Next, we propose an improved MBF calculation approach to alleviate some of those confounders, to reduce the bias, and, potentially in extension, help to improve the reproducibility of Flow‐sensitive Alternating Inversion Recovery (FAIR) myoASL. Namely, subject‐specific blood $T_{1}$ relaxation times and, for spGRE readouts only, additional saturation‐prepared baseline acquisitions are used to calculate MBF. Finally, the repeatability of myoASL with and without corrections is studied in healthy volunteers.

## 2 THEORY

### 2.1 ASL signal model

**FIGURE 1:**
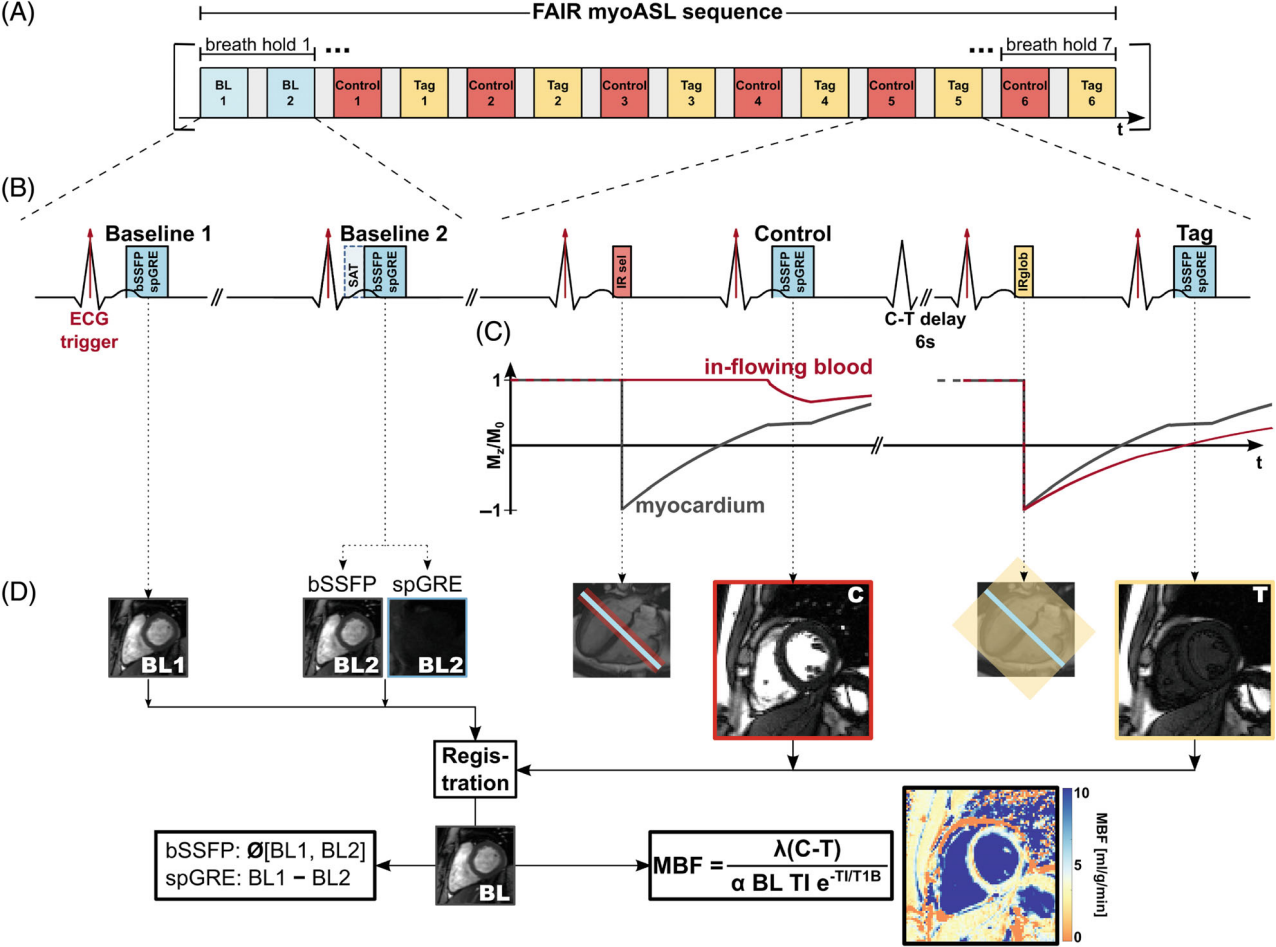
(A, B) Sequence diagram of the FAIR‐myoASL sequence and (D) processing pipeline used in this study. (C) Temporal evolution of the longitudinal magnetization after an initial inversion pulse and during the imaging readout in the subsequent heartbeat.

MBF quantification in myoASL is most commonly based on Buxton's general kinetic model (GKM).^16^ In the GKM, the difference between control and tag signal is modeled based on the transport of inverted magnetization into the imaging volume with arterial blood. The present work focuses on a FAIR‐ASL sequence (Figure 1), for which the GKM can be derived as:

(1) $$\text{MBF}=\frac{\lambda (I_{C}-I_{T})}{\delta I_{\text{BL}}\text{TI}e^{-\text{TI}/T_{1,B}}}\text{,}$$

with control ($I_{C}$), tag ($I_{T}$), and baseline signal ($I_{\text{BL}}$), inversion time TI, inversion efficiency $\delta =1-\cos(\alpha_{\text{inv}})$, blood‐water partition coefficient λ=1mL/g,^20^, ^21^ and blood $T_{1}$ relaxation time $T_{1,B}$. Due to the substantially faster flow in the heart compared to other anatomies, the labeling slab is considered to be small relative to the fast flow in coronary arteries during the TI.^17^ Therefore, as previously applied in cardiac ASL,^17^, ^22^ in the present work it has been decided to neglect the effect of the ATT in the model as a first approximation.

### 2.2 Magnetization modulation function

Imaging in myoASL has been previously proposed with bSSFP or spGRE snapshot readouts. These readouts lead to a significant modulation of the magnetization, which is expressed as a magnetization modulation function (MMF, $f_{\text{MMF}}$) throughout this work.

The signal equations for bSSFP and spGRE readout are provided in Appendix A. They can be simplified in the form of a general affine linear model for the MMF:

(2) $$f_{\text{MMF}}(x)=Ax+B\text{.}$$

Here, the coefficients A and B depend on the acquisition parameters as well as $T_{1}$ and $T_{2}$, while $x=M_{z}(t_{0})$ represents the initial magnetization immediately prior to the readout. Due to the low systolic coronary blood flow,^23^, ^24^ in‐ and out‐flow effects during the image readout were considered negligible and, thus, were not explicitly considered in the MMF. Based on the MMFs in the Appendix (Equations (A1), (A5)), A and B are given as

(3) $$\begin{array}{ll} A=\left\{\begin{array}{ll} \sin(\frac{\alpha }{2}){\left(E_{1}\cos^{2}(\frac{\alpha }{2})+E_{2}\sin^{2}(\frac{\alpha }{2})\right)}^{n},\; & \text{bSSFP}\\ \rho \cos^{n}(\alpha )E_{1}^{n-1},\; & \text{spGRE} \end{array}\right. & \end{array}$$

and

(4) $$\begin{array}{ll} B=\left\{\begin{array}{ll} (1-{\left(E_{1}\cos^{2}(\frac{\alpha }{2})+E_{2}\sin^{2}(\frac{\alpha }{2})\right)}^{n})M_{ss},\; & \text{bSSFP}\\ \frac{1-{\left(\cos(\alpha )E_{1}\right)}^{n-1}}{1-\cos(\alpha )E_{1}}(1-E_{1})\cos(\alpha )\rho M_{z,eq},\; & \text{spGRE} \end{array}\right. & \end{array}$$

with $E_{1/2}=e^{-TR/T_{1/2}}$, proton density ρ, steady‐state and equilibrium longitudinal magnetization $M_{ss}$ and $M_{z,\text{eq}}$, flip angle (FA) α, and n applied imaging pulses.

#### 2.2.1 FAIR‐myoASL sequence

In a FAIR‐myoASL measurement, the imaging signal can be modeled with blood ($I_{B}$) and myocardial contributions ($I_{M}$) weighted by the blood‐volume‐fraction $V_{B}$ and its complement $V_{M}=1-V_{B}$, respectively. Due to differences in the relaxation times,^25^, ^26^ the coefficients $A_{M}$ and $A_{B}$ in the MMF (Equations 3and 4) differ between $I_{B}$ and $I_{M}$.

Using the approach of Buxton's GKM (Equation 1) and the image signals as derived in the Appendix (Equations (B2), (B3), (B4), (B5), (B6), (B7), (B8), (B9)), the ratio of control, tag, and baseline signal can be given as:

(5) $$\frac{I_{C}-I_{T}}{I_{\text{BL}}}=\frac{V_{B}f_{\text{in}}A_{B}(x_{B}^{+}-x_{B}^{-})}{V_{M}(A_{M}x_{M}^{+}+B_{M})+V_{B}(A_{B}x_{B}^{+}+B_{B})}\text{.}$$

This relation only yields the unbiased, true perfusion rate $f_{\text{in}}$, in the case of $A_{B}=A_{M}=1$ and $B_{B}=B_{M}=0$ which is implicitly assumed in Buxton's GKM. In an experimental setting, however, this condition is not met due to the long echo trains (n≫1) in particular for snapshot readouts. Hence, the obtained perfusion rate is confounded by acquisition parameters such as the FA and the acquisition matrix size (AMS), which determines the number of imaging pulses applied prior to the k‐space center.

**FIGURE 2:**
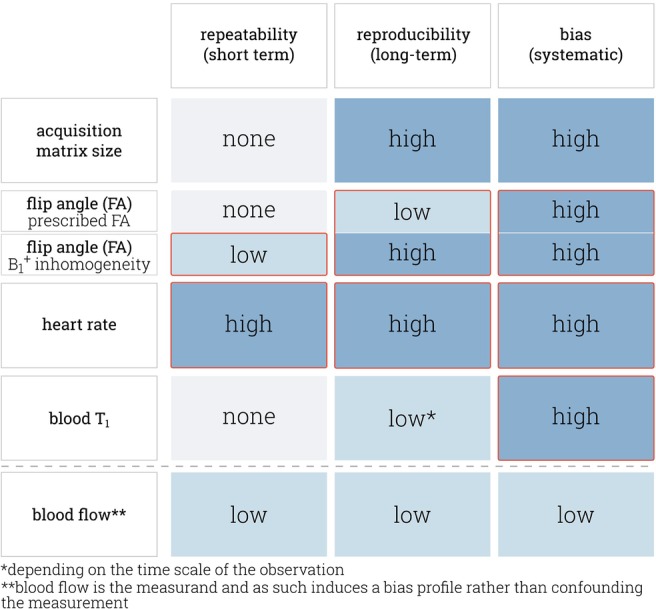
Various acquisition‐related (acquisition flip angle and matrix size) and physiological parameters (heart rate, blood $T_{1}$, blood flow) influence the precision (repeatability, reproducibility) and accuracy (bias) of FAIR‐myoASL measurements. Except for blood flow effects during image readout, these factors were investigated in this work. Parameters affected by the proposed correction approach are framed in red. Repeatability is mainly influenced by physiological noise, while reproducibility relates to variability on longer time scales and between systems/set‐ups. Bias relates to intrinsic systematic deviations and is a common source of lack of reproducibility.^38^ The different factors manifest as different types of myocardial blood flow (MBF) errors depending on their respective time scale: Different prescribed matrix sizes and flip angle values in separate measurements as well as subject‐specific $B_{1}^{+}$ field distributions impart different biases and impair reproducibility.^46^, ^61^ Further, a varying heart rate (HR) can cause changes in the coronary blood flow^62^—a major source of physiological noise—as well as in the sequence timing, which confounds MBF values and renders them subject to different biases. Repeatability and reproducibility can further be compromised by changes in the HR across control‐tag pairs or across measurements/subjects, respectively. As the value of $T_{1,B}$ depends on numerous physiological factors which might change over longer periods of time, this can affect the MBF bias and reproducibility. Finally, replacement of spins due to flow‐effects can alter the magnetization modulation function in FAIR‐myoASL, potentially imparting bias and compromising reproducibility.

These acquisition parameters can, thus, influence the precision and accuracy of the measurement. Figure 2 illustrates the interdependencies for a selection of parameters relevant to this study, namely: AMS, acquisition FA, heart rate variations, $T_{1,B}$, and blood flow. Most of these factors, such as the FA and AMS, affect the accuracy and might impart bias on FAIR‐myoASL‐based MBF. However, because these parameters might vary on different time scales, they can also compromise the reproducibility and even repeatability of FAIR‐myoASL.

#### 2.2.2 Saturation‐baseline

As apparent from Equation (5), eliminating the coefficients $B_{B}$ and $B_{M}$ from the image signal can reduce the dependence on acquisition parameters. This can be achieved with the acquisition of an additional saturation‐prepared baseline image ($I_{\mathrm{BL},\mathrm{Sat}}$), such that the signal only represents the imaging readout and not the magnetization history anymore. To this end, a saturation prepulse immediately prior to the baseline image can be used. Assuming perfect saturation, the initial magnetization of both myocardium and blood can be considered to be zero ($x_{M}=x_{B}=0$) for the saturation‐baseline signal. If the signal difference between the saturation and original baseline (see Equation B10) is used instead of the original baseline signal in Equation (5), the ratio of control and tag image becomes:

(6) $$\begin{array}{ll} \frac{I_{C}-I_{T}}{I_{\text{BL}}-I_{\mathrm{BL},\mathrm{Sat}}} & =\frac{V_{B}f_{\text{in}}A_{B}(x_{B}^{+}-x_{B}^{-})}{V_{M}A_{M}x_{M}^{+}+V_{B}A_{B}x_{B}^{+}}=\\ & =\frac{V_{B}f_{\text{in}}(x_{B}^{+}-x_{B}^{-})}{V_{M}\frac{A_{M}}{A_{B}}x_{M}^{+}+V_{B}x_{B}^{+}}\text{.} \end{array}$$

Due to the different MMFs, this leads to different factors $\frac{A_{M}}{A_{B}}$ in Equation (6) for the two readout types:

(7) $$\begin{array}{ll} \frac{A_{M}}{A_{B}}=\left\{\begin{array}{ll} {\left(\frac{E_{1,M}\cos^{2}(\frac{\alpha }{2})+E_{2.M}\sin^{2}(\frac{\alpha }{2})}{E_{1,B}\cos^{2}(\frac{\alpha }{2})+E_{2,B}\sin^{2}(\frac{\alpha }{2})}\right)}^{n},\; & \text{bSSFP}\\ e^{-(n-1)\cdot TR\cdot (\frac{1}{T_{1,M}}-\frac{1}{T_{1,B}})},\; & \text{spGRE} \end{array}\right.. & \end{array}$$

Notably, the use of the saturation‐baseline eliminates the FA dependence for the case of spGRE readout and the only residual acquisition parameter related influence is given by the AMS n. With bSSFP readout, however, the signal ratio remains both FA and AMS dependent as the transverse magnetization contributes to the readout signal at each TR.

## 3 METHODS

### 3.1 MyoASL sequence

Based on the considerations above, a double ECG‐triggered FAIR‐ASL sequence building on the design by Do et al. ^22^, ^27^ is proposed. As depicted in Figure 1, nonlabeled control and labeled tag images are acquired in an alternating fashion. For the control image, a spatially selective, adiabatic inversion pulse is applied in one heartbeat. The image acquisition is performed in the subsequent heartbeat during the same cardiac phase. To ensure consistent inversion within the imaging slice, the inversion slab is chosen three times as thick as the imaging slice. Following a 6 s long delay, the tag image is acquired in the same fashion but using a non‐selective adiabatic inversion pulse. Each myoASL measurement comprised six pairs of control and tag images, referred to as individual scans, using either bSSFP or spGRE readouts.

Additionally in each measurement, a pair of baseline images was acquired without preceding inversion pulses. For bSSFP readouts, both baseline images are acquired without any preparation pulses, while for spGRE an additional saturation prepulse is added immediately prior to the readout of the second baseline image.

Postprocessing of images including MBF quantification and statistical analysis was performed in MATLAB (MathWorks). The MBF was quantified using Buxton's GKM as described in Equation (1). For bSSFP‐based MBF calculation, the baseline signal $I_{\text{BL}}$ corresponds to the average of the two baseline images. With spGRE readout, the saturation‐baseline image $I_{\mathrm{BL},\mathrm{Sat}}$ is subtracted from the original one and the difference image is used as the baseline value in MBF calculations as given in Equation (6). As shown in Equation (7), this saturation‐baseline correction does not eliminate FA dependencies in bSSFP readouts and is therefore not applied for those. The double ECG‐triggering of both labeling pulses and image readouts leads to a variable, heart rate dependent TI. For MBF calculation, TI was evaluated using either individual TIs, an average inversion time $\bar{TI}$ for each control‐tag pair, or a global TI averaged per sequence.

In previous studies on cardiac ASL, $T_{1,B}$ was set to a fixed, literature based value between 1650 and 1700 ms.^13^, ^22^, ^28^, ^29^ To avoid discrepancies with the actual $T_{1}$, subject‐specific $T_{1}$ relaxation times are used in a second quantification method.

In summary, perfusion values were calculated in four different modes depending on the readout:

- bSSFP readout with conventional, uncorrected MBF calculation (fix $T_{1,B}$, no saturation‐baseline)
- bSSFP readout with corrected MBF calculation (measured individual $T_{1,B}$, no saturation‐baseline)
- spGRE readout with conventional, uncorrected MBF calculation (fix $T_{1,B}$, no saturation‐baseline)
- spGRE readout with corrected MBF calculation (measured individual $T_{1,B}$ and saturation‐baseline)

Based on previous FAIR‐myoASL studies,^13^, ^27^, ^29^ the uncorrected MBF calculation from bSSFP‐images is considered as the reference configuration throughout the remainder of this work.

### 3.2 Imaging

All imaging was performed at 3T. In all experiments, a WET saturation pulse^30^, ^31^ has been used for preparation of the saturation‐baseline. The detailed sequence parameters for all experiments are provided in Table 1.

**TABLE 1:** Sequence parameters of the FAIR‐myoASL sequence for phantom and in vivo measurements.

	FA (°)		TE/TR (ms)						
Experiment	bSSFP	spGRE	bSSFP	spGRE	Matrix size	FOV (mm 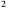 )	HR (bpm)	Resolution/ slice thickness	Partial Fourier/ generalized auto‐calibrating partially parallel acquisition rate
Phantom – Varying HR	70	15	1.6/3.3	3.3/6.3	160 × 160	280 × 280	40–120		
Phantom – Varying FA	1–80	1–40	1.6/3.3	3.3/6.3	160 × 160	280 × 280	60	1.7 × 1.7 mm 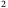	6/8
					208 × 208	364 × 364		8 mm	*R* = 2
In vivo	70	18	1.2/2.4	1.9/2.9	154 × 192	272 × 340	n/a		

Abbreviations: FA, flip angle; FOV, field of view; HR, heart rate; spGRE, spoiled GRE; TE, echo time; TR, pulse repetition time.

#### 3.2.1 Phantom experiments

A phantom comprising 13 NiCl_2_‐doped agarose vials submerged in agarose gel was used, with $T_{1}$ relaxation times ranging between 1100 and 2500 ms and $T_{2}$ relaxation times between 50 and 170 ms. For further evaluation, five vials with relaxation times in the physiological range were selected. Phantom experiments were performed at 3T (Magnetom Skyra, Siemens Healthineers). The FAIR‐myoASL sequence was used with the acquisition parameters provided in Table 1.

Three sets of experiments were performed to investigate the effect of physiological and acquisition parameters, respectively. First, phantom data were acquired for both readout types for a range of FAs in bSSFP and spGRE with two matrix sizes (i.e., with different AMS). Further, images were acquired with varying simulated HR which resulted in varying TI and, lastly, with fixed HR, FA and AMS for control‐tag delays between 6 and 12 s.

Prior to further processing, the signal polarity had been restored based on the recovery curves obtained with different TIs.^32^ To simulate the effect of flow during TI, a blood volume fraction of 0.14^33^ and a blood replacement/in‐flow rate of 0.29 1/s were simulated, resulting in an effective MBF input value of 2.4 mL/g/min. The assumed in‐flow rate corresponds to about 4 mL/s for a myocardial blood volume of 15 mL (about 10% of the left‐ventricular mass^34^, ^35^). Following Equations (B7)–(B9), the control, tag and baseline signals were generated from the image signal of different vials, which was obtained from manually drawn ROIs. The inverted signal contributions to $I_{C}$ and $I_{T}$ were taken from the selective and non‐selective inversion recovery, respectively. For the myocardial signal, a vial with $T_{1}$/$T_{2}$ relaxation time of 1460/45 ms was used. The blood signal was taken from four different vials with $T_{1}$ relaxation times of 1770–2300 ms and $T_{2}$ relaxation times of 45–124 ms.

In addition to the phantom experiments, numerical simulations have been performed to assess the effect of the same physiological and acquisition‐related parameters on myoASL‐based MBF. Details can be found in the Numerical Simulations section of the Appendix S1.

#### 3.2.2 In vivo experiments

The present study was approved by the local institutional review board and written informed consent was obtained from all participants prior to examination. Nine healthy subjects (3 female, 6 male, 36±8 years) with no history or current symptoms of cardiovascular disease were included in this study. The in vivo scans were performed at 3T (Magnetom Prisma, Siemens Healthineers).

MOLLI^32^ $T_{1}$ maps were acquired in each subject to obtain blood $T_{1}$ ($T_{1,B}$) times in the corrected MBF calculation. In the individual $T_{1}$ maps, an ROI was manually drawn in the left ventricle and $T_{1,B}$ was determined as the mean value across all pixels within this ROI. For FAIR‐myoASL, labeling and imaging were placed in the systole for increased perfusion signal.^28^ The detailed imaging parameters are given in Table 1. In six out of the nine subjects, two repetitions of two FAIR‐myoASL sequences (bSSFP and spGRE) were acquired. Images were acquired during 12–16 s long breath‐holds, depending on the subject's heart rate, with one image pair (baseline/control‐tag) per breath‐hold.

Each FAIR‐myoASL sequence consisted of seven breath‐holds: one for the baseline images and six for the six control‐tag image pairs. The bSSFP and spGRE data sets were group‐wise registered for each subject.^36^ Subsequently, control‐tag pairs subject to ECG mis‐triggering or a difference in TI larger than approximately 120 ms were excluded prior to image analysis. For each subject, the myocardium as well as a septal ROI were segmented manually.^37^ Pixel‐wise perfusion maps and segment‐wise septal MBF were obtained using uncorrected calculation in bSSFP and spGRE as well as corrected spGRE calculation as described above. Global MBF values are reported as the mean MBF across the myocardial ROI and across all control‐tag image pairs in each repetition. Mean septal MBF values are reported as the septal MBF averaged across all control‐tag image pairs.

#### 3.2.3 Statistical analysis

In simulation and phantom experiments, the correlation of MBF with HR and FA was assessed using Spearman's correlation, respectively. To further evaluate the HR and FA dependence, slope and intercept values were obtained from a linear regression of simulation and phantom MBF. Moreover, the relative MBF error $\left(\frac{{\text{MBF}}_{\text{phantom}}-{\text{MBF}}_{\text{true}}}{{\text{MBF}}_{\text{true}}}\right)$ was compared across the calculation modes using a Friedman test for group‐wise comparison, followed by a Wilcoxon signed‐rank test for pair‐wise comparison. For in vivo septal MBF and each readout‐calculation combination, the intra‐subject variability within a measurement $\bar{\sigma_{p}}$ was calculated as the PN averaged across all subjects:

(8) $$\bar{\sigma_{p}}=\frac{1}{N_{S}}\overset{N_{S}}{\underset{j=1}{\sum }}{\text{PN}}_{j}\text{,}$$

with number of subjects $N_{S}=9$. The PN for each repetition m with $N_{CT}^{j,m}$ control‐tag image pairs is obtained as^13^

(9) $$\begin{array}{ll} {\text{PN}}_{j,m} & =\frac{1}{\sqrt{N_{CT}^{j,m}}}\sigma {\left({\text{MBF}}_{j,m}\right)}_{N_{CT}^{j,m}}\\ & =\sqrt{\frac{\sum_{i=1}^{N_{CT}^{j,m}}{\left({\text{MBF}}_{i,j,m}-\bar{{\text{MBF}}_{j,m}}\right)}^{2}}{N_{CT}^{j,m}(N_{CT}^{j,m}-1)}}. \end{array}$$

The mean between‐measurement, intra‐subject variability $\bar{wsSD}$ of each sequence was defined as the difference in mean MBF from the two repetitions scaled by $\sqrt{2}$ and averaged over the corresponding subcohort ($N_{S}=6$):^38^

(10) $$\begin{array}{ll} \bar{wsSD} & =\frac{\sum_{j=1}^{N_{S}}wsSD_{j}}{N_{S}}\\ wsSD_{j} & =\frac{\left|{\bar{\text{MBF}}}_{j,1}-{\bar{\text{MBF}}}_{j,2}\right|}{\sqrt{2}}. \end{array}$$

Lastly, the inter‐subject variability isSD was evaluated as the SD across the individual mean MBF values:

(11) $$\begin{array}{ll} isSD & =\sqrt{\frac{\sum_{j=1}^{N_{S}}{\left({\bar{\text{MBF}}}_{j}-\bar{\text{MBF}}\right)}^{2}}{N_{S}-1}}\\ \bar{\text{MBF}} & =\frac{1}{N_{S}}\overset{N_{S}}{\underset{j=1}{\sum }}{\bar{\text{MBF}}}_{j}. \end{array}$$

In subjects with multiple repetitions, only MBF data from the first repetition has been used to obtain $\bar{\sigma_{p}}$ and isSD. The intra‐ and inter‐subject variability were compared across the calculation modes using a Friedman test for group‐wise comparison, followed by a Wilcoxon signed‐rank test for pair‐wise comparison. A significance level of 0.05 was used in all statistical tests.

## 4 RESULTS

### 4.1 Phantom results

Simulated perfusion showed negligible differences whether calculated with individual TIs, an average inversion time $\bar{TI}$ for each control‐tag pair, or a global TI averaged per sequence as shown in Figure S1. Therefore, in all further phantom and in vivo measurements MBF was calculated with a pairwise averaged $\bar{TI}$ for each control‐tag pair. While differently evaluated TIs in MBF calculation led only to small changes in the MBF deviation, the HR variability appeared as a major confounder in myoASL‐MBF.

The following phantom results are shown for all four combinations of readout and MBF calculation. Here, corrected calculation in bSSFP refers to using individual $T_{1,B}$ values, but no saturation‐baseline which is only applied for spGRE as explained in the Section 3. The relative error in simulated and phantom MBF for varying control‐tag delays is shown in Figure S2. For uncorrected as well as corrected calculations, the MBF values from bSSFP and spGRE readouts were largely constant over the range of applied control tag delays. The difference in MBF between a 6 s long delay and the steady state was <2.2%/<6.0% (bSSFP/spGRE) in simulations and <4.8%/<3.8% (bSSFP/spGRE) in phantom experiments. Thus, a control‐tag delay of 6 s was chosen for all further experiments.

#### 4.1.1 Flip angle

**FIGURE 3:**
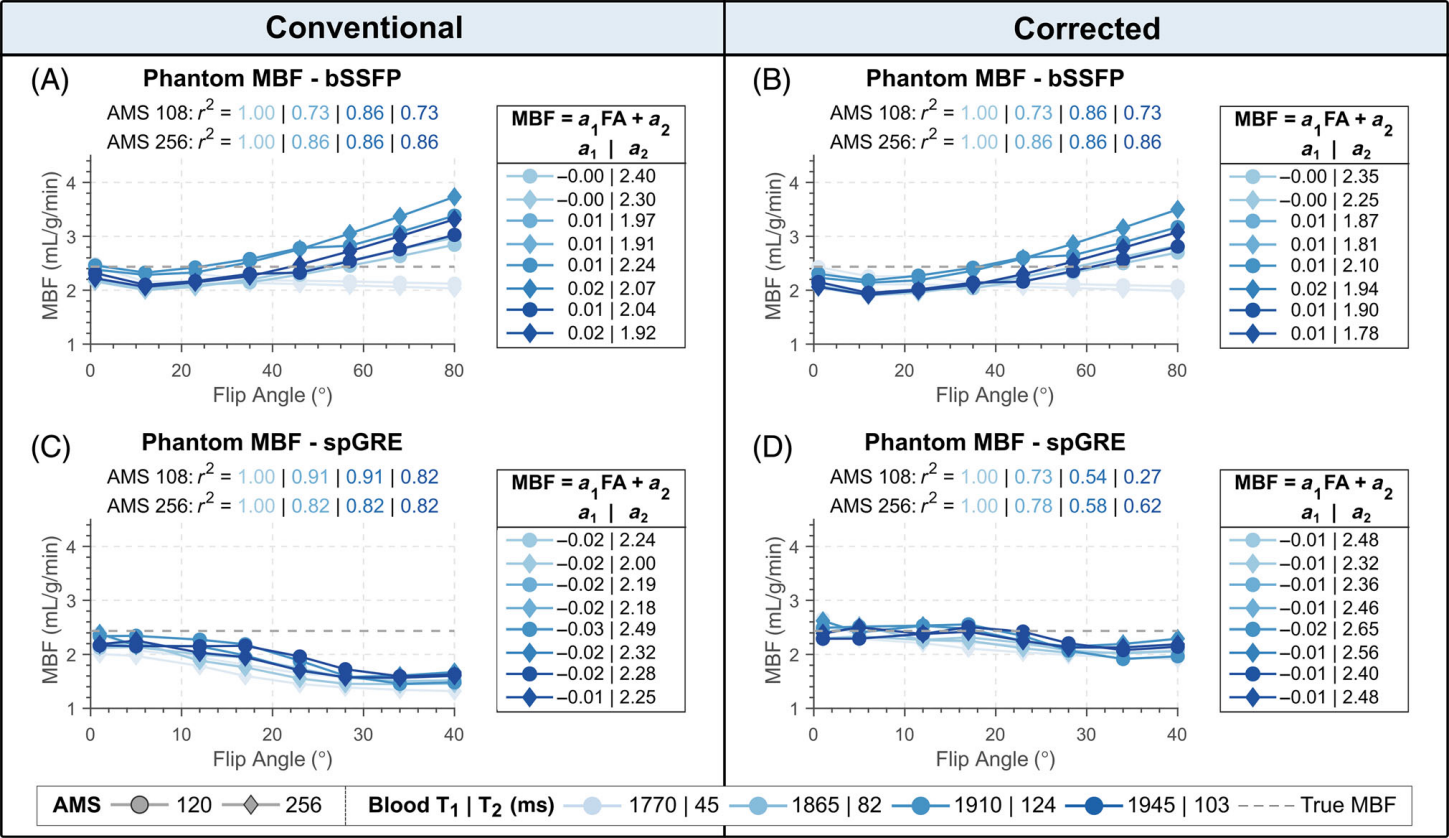
Phantom myoASL‐MBF from bSSFP and spoiled GRE (spGRE) readouts. Myocardial blood flow (MBF) was calculated with (A), (C) fixed and (B), (D) individual blood $T_{1}$ ($T_{1,B}$). Additionally, for corrected spGRE (D) the saturation‐baseline approach as proposed in this work was used in MBF calculation. MBF is shown as a function of acquisition flip angle (FA) for two acquisition matrix sizes (AMS) and four phantom vials (i.e. different blood $T_{1}$ and $T_{2}$). The slope ($a_{1}$) and intercept ($a_{2}$) for each vial are obtained from linear regression. Across all vials, a strong FA dependence of bSSFP‐ and spGRE‐based MBF is observed and this effect is exacerbated for larger AMS. When the proposed correction is used in spGRE readouts, the FA dependence is alleviated over the range of acquired FAs.

Figure 3 shows the phantom MBF plotted against the acquisition FA for two different AMSs. In uncorrected bSSFP, phantom MBF increased with increasing FA for all vials ($0.73<R^{2}<0.86$, slope: 0.01–0.02) except one ($T_{1}$/$T_{2}$ of 1770/45 ms), where MBF was underestimated with increasing FA ($R^{2}=1$, slope: −0.004). For all vials, longer AMS resulted in increased MBF values. This FA dependence remained for bSSFP readout when MBF was calculated with $T_{1,B}$‐correction ($0.73<R^{2}<1$). In uncorrected spGRE, MBF values correlated strongly with FA ($0.82<R^{2}<1$). Phantom MBF decreased with increasing FA and was lower for longer AMS for all vials, with linear slopes of −0.017 to −0.027. Using corrected calculation, spGRE‐based MBF stayed largely constant around 2.48 mL/g/min up to about 25° from where it decreased slightly to 2.14 mL/g/min ($0.27<R^{2}<1$, slope: −0.006 to −0.010). For all vials, group‐wise comparison revealed a significant difference in relative MBF error among the compared readout‐calculation combinations (p<0.05). With fully corrected calculation in spGRE‐readouts, the relative MBF error was significantly reduced compared to uncorrected spGRE (p<0.05) in all vials and showed a small, non‐statistically significant reduction compared to uncorrected bSSFP (0.05<p<0.25) in all vials except one (p=0.74/0.84 for AMS 120/256, $T_{1}$/$T_{2}$ of 1865/82 ms). Thus, the saturation‐baseline approach was used for the correction of spGRE readouts in the remainder of this work.

Phantom‐based MBF values from different vials are plotted as a function of simulated HR in Figure S3. If an incorrect $T_{1,B}$ was used for quantification, phantom MBF showed a weak dependency on the HR (on average 0.01 mL/g/min per 100 ms change in RR). This effect was more pronounced with larger difference between actual and quantification $T_{1,B}$ (1700 ms). Significant differences in relative MBF error were observed among the three readout‐calculation combinations when examined through group‐wise comparison (p<0.05). When the correct $T_{1,B}$ was used, the relative MBF error was significantly reduced compared to uncorrected MBF calculation (p<0.05) and MBF values were largely constant with HR for both readout types (0.03$<R^{2}<$0.21/0.30$<R^{2}<$0.64 bSSFP/spGRE). A bias in MBF of 0.43/0.16 mL/g/min (bSSFP/spGRE) remained across the different $T_{1}$/$T_{2}$ values.

Simulated MBF from bSSFP‐readouts was overestimated with increasing FA and AMS whether calculated with or without individual $T_{1,B}$ (slope: 0.02–0.06, $R^{2}=1$), as depicted in Figure S4. For the case of uncorrected spGRE, MBF was largely constant up to FAs of about 5°, and was increasingly underestimated with FA increasing beyond 5° (slope: −0.08 to −0.07), $R^{2}=1$). With fully corrected calculation, spGRE‐based MBF was largely constant over the entire range of FAs (AMS 120: slope 0.0, $0.0<R^{2}<0.30$; AMS 256: slope 0.0–0.01, $0.11<R^{2}<0.44$). As shown in Figure S5, simulated spGRE‐based MBF was constant over the range of simulated blood $T_{2}$ values, while bSSFP‐based MBF showed a strong nonlinear relation. MBF obtained with bSSFP and spGRE readouts showed a moderate dependence on $T_{1,B}$, which is eliminated when calculated with the correct $T_{1,B}$. Increasing measurement errors in $T_{1,B}$ led to increasing MBF errors (approximately 3% per 100 ms) for all four combinations of readout and calculation mode (Figure S6). Further, if an inaccurate $T_{1,B}$ is used in quantification, MBF shows a weak HR dependence in both readouts, as illustrated in Figure S7, which is alleviated when calculated with true $T_{1,B}$.

### 4.2 In vivo results

**FIGURE 4:**
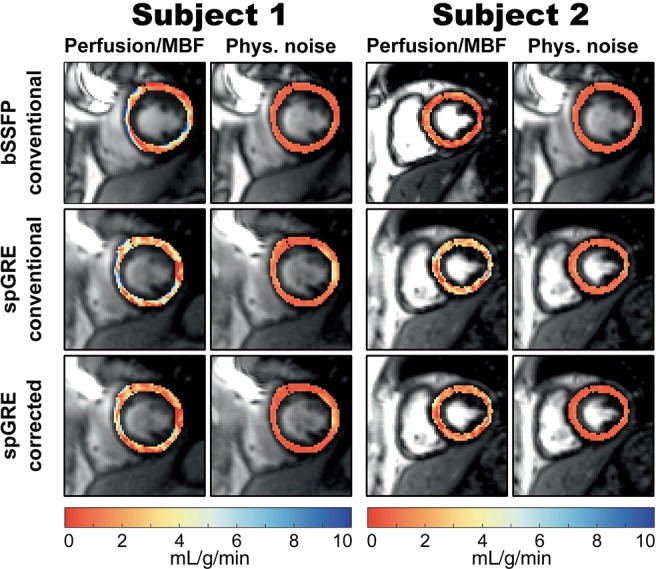
MyoASL‐perfusion and physiological noise (PN) maps for two representative subjects. For uncorrected bSSFP/spGRE readouts, mean global myocardial blood flow (MBF) ± PN was 3.05 ± 0.76/3.14 ± 1.52 mL/g/min in subject 1 and 0.75 ± 0.34/2.63 ± 1.36 mL/g/min in subject 2. Uncorrected bSSFP‐maps appear visually more homogeneous and show lower PN across the myocardium compared to uncorrected spGRE‐maps. With the proposed correction, however, spGRE‐maps were on par with uncorrected bSSFP‐maps showing improved image quality and reduced PN compared to uncorrected spGRE. Mean global MBF ± PN in this case was 1.98 ± 0.96 mL/g/min in subject 1 and 1.67 ± 0.87 mL/g/min in subject 2.

Based on the relatively mild effect of $T_{1,B}$ compared to the FA on simulated and phantom MBF, in vivo results from bSSFP readouts are presented with uncorrected MBF calculation only. Over all subjects, mean blood $T_{1,B}$ was 1860±68 ms and the HR ranged from 47 to 72 bpm. Perfusion maps and corresponding PN maps of the myocardium are shown for two representative subjects in Figure 4. For uncorrected bSSFP, mean global MBF±PN were 3.05 ± 0.76 mL/g/min and 0.75 ± 0.34 mL/g/min for the two subjects, respectively. In spGRE, global MBF was 3.14 ± 1.52/2.63 ± 1.36 mL/g/min (subject 1/2) with uncorrected and 1.98 ± 0.96/1.67 ± 0.87 mL/g/min (subject 1/2) with fully corrected calculation. In visual assessment, uncorrected bSSFP‐based maps appeared more homogeneous compared to uncorrected spGRE‐based maps. With corrected MBF calculation, however, the image quality of spGRE‐based perfusion maps was improved compared to the uncorrected spGRE‐maps and visually comparable to the conventional bSSFP approach.

**FIGURE 5:**
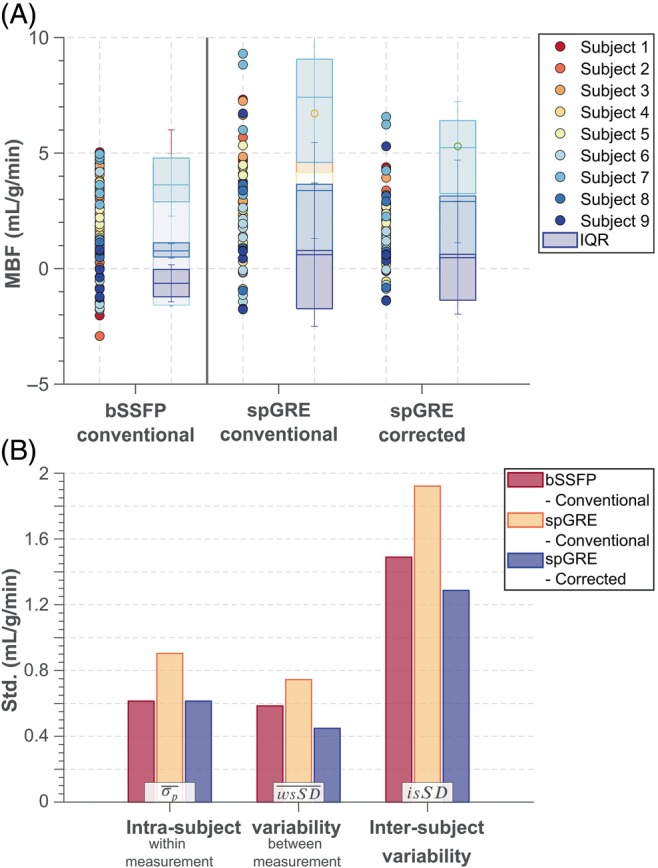
(A) In vivo septal myoASL‐MBF for all acquired control‐tag pairs and all nine subjects. With uncorrected calculation, myocardial blood flow (MBF) from spGRE readouts showed larger variation compared to bSSFP‐based MBF. When MBF was calculated with the proposed correction, however, the spread in spGRE‐MBF values was reduced compared to both uncorrected bSSFP and spGRE. This is also reflected in the (B) mean intra‐ and inter‐subject variability: With the proposed correction, spGRE readouts show improved reproducibility compared to uncorrected spGRE and are comparable to uncorrected bSSFP.

The intra‐subject variability within ($\bar{\sigma_{p}}$) and between measurements ($\bar{wsSD}$) as well as the inter‐subject variability (isSD) based on septal MBF are displayed in Figure 5 for the three different combinations of readout and MBF calculation. In group‐wise comparison, $\bar{\sigma_{p}}$ and $\bar{wsSD}$ showed significant differences among the three combinations of readout and calculation mode (p<0.05). Mean within‐measurement, intra‐subject variability was lower in uncorrected bSSFP (0.61 mL/g/min) than in uncorrected spGRE (0.90 mL/g/min, p=0.30). The mean within‐measurement, intra‐subject variability in corrected spGRE‐based MBF calculation (0.60 mL/g/min) was on par with uncorrected bSSFP (p=0.73). Uncorrected bSSFP showed lower $\bar{wsSD}$ (0.58 mL/g/min, p=0.44) and isSD (1.49 mL/g/min) compared to uncorrected spGRE (0.74 and 1.92 mL/g/min, respectively). However, when spGRE‐MBF was calculated with individual $T_{1,B}$ and saturation‐baseline, $\bar{wsSD}$ was reduced compared to uncorrected spGRE by 40% (p<0.05) and showed a slight but not significant reduction compared to uncorrected bSSFP 22% (p=1.0). With fully corrected MBF quantification, the isSD of spGRE‐MBF was reduced compared to uncorrected bSSFP/spGRE by 13%/33%, respectively.

## 5 DISCUSSION

In this work, we investigated how physiological and acquisition‐related parameters affect FAIR‐myoASL‐based MBF measurements, when bSSFP or spGRE readouts are used. Our simulation and phantom experiments suggest that, out of the investigated parameters, the acquisition FA has the strongest effect on the MBF and may cause spurious MBF deviations. Through an adapted baseline acquisition this effect can be mitigated for spGRE readouts. Furthermore, inaccurate blood $T_{1}$ relaxation times in the MBF calculation led to a mild HR dependence which can be reduced if calculated with individual $T_{1,B}$. Using both approaches, spGRE‐MBF measurements with increased reproducibility have been obtained.

This study uses the Buxton GKM which is a common choice with cardiac FAIR‐ASL.^13^, ^17^, ^22^ However, several simplifications are made when applying this kinetic model. Importantly, the arterial transit time (ATT) effect is considered to be negligible due to the relatively small size of the labeling slab compared to the relatively fast flow in the coronary arteries. However, ATT is known to be a major confounder to accurate perfusion measurements in other anatomies,^39^ and the validity of this simplification in cardiac applications warrants thorough investigation. Alternative approaches, such as saturation preconditioning of the signal preceding the bolus edge,^40^ or velocity selective labeling,^41^, ^42^ where labeling and imaging volumes coincide, are promising for mitigating this confounder. Furthermore, measurements, with multiple postlabeling delays may allow for joint quantification of the ATT to fully correct for this effect.^43^ These approaches and the meteorological characterization of the impact of ATT on FAIR‐based cardiac ASL quantification remain an important topic of future studies.

In this study, imaging is performed during systole, when blood flow is minimal,^23^, ^24^ resulting in less than 1% of spins being exchanged throughout the imaging readout. Consequently, the MMF is only very weakly affected by flow and spin‐exchange during the acquisition readout. However, in approaches that involve extended imaging readouts during diastole or continuous imaging readouts, like “cine‐ASL”^44^, the in‐flow effect becomes more relevant. Recently proposed numerical models aim to capture this phenomenon,^45^ offering a promising avenue for future research in cardiac ASL. Additionally, the proposed correction scheme assumes that the MMF is identical for baseline, control and tag acquisitions. While this is a common assumption in all ASL techniques,^16^ differences in MMF among those images may be caused by factors such as changes in the in‐flow rate during the readout or changes in the effective FA. To that end, repeating baseline acquisitions throughout the measurement may be useful to minimize the resulting variability in perfusion calculation.

The acquisition FA was identified as a strong confounder in simulated and phantom myoASL‐MBF measurements. In both simulation and phantom experiments, bSSFP‐based MBF increased with increasing FA while spGRE‐based MBF decreased with increasing FA, when conventionally calculated. As a result of the increased number in applied RF pulses, the effect of the imaging readout is exacerbated for larger AMS used in a snapshot readout. These results are especially relevant in view of the high $B_{1}^{+}$ variability across the myocardial region. Particularly at 3T, variations of up to 50% of the nominal FA have been observed.^46^ In order to alleviate this FA dependence, we proposed an adapted baseline acquisition and MBF calculation for spGRE readouts using an additional saturation‐baseline image. Due to the nature of the MMF in bSSFP, this approach can only correct for FA effects with spGRE readouts. However, in this case, the FA dependence is fully eliminated in simulated MBF and substantially reduced in phantom experiments, potentially alleviating a major acquisition‐related confounder.

Perfusion values were comparable between bSSFP and spGRE readouts in simulation and phantom experiments. However, bSSFP‐based MBF showed a larger variability with blood $T_{1}$/$T_{2}$ relaxation times compared to spGRE due to the $T_{2}$ dependence of the bSSFP readout signal. Our simulation and phantom results further show that a mismatch between true and quantification $T_{1,B}$ may render myoASL‐MBF mildly dependent on HR. Cardiac ASL has previously been reported in the literature with intra‐subject variability between 7.5%^27^ and 28%.^28^ In the present study, those values ranged between 26% and 39%. Thus, acquisition‐related factors, such as FA and AMS, can have a relevant impact on the measurement error in cardiac ASL (up to 60% of MBF). HR‐related factors, on the other hand, were found to be mostly negligible in our results (up to 2% of MBF). As a result, the effect of using individual $T_{1,B}$ to alleviate the HR dependence is less noticeable in visual assessment compared to using the saturation‐baseline for FA correction. Nonetheless, in light of the prevalence of $T_{1}$ mapping in clinical practice,^47^ individual $T_{1,B}$ values can easily be obtained in common CMR examinations, and can often be incorporated without adding extra scans to the protocol.

In vivo, mean global MBF values from uncorrected bSSFP readout (2.10 ± 0.95 mL/g/min) agreed with previously reported PET‐based resting MBF (0.74‐2.43 mL/g/min^48^). Compared to the reported MBF at rest in healthy subjects as obtained from first‐pass perfusion MRI (0.62 ± 0.13 to 1.24 ± 0.19 mL/g/min^49^, ^50^), the observed myoASL‐MBF values were elevated across all readout and calculation mode combinations. However, previous studies using myoASL reported values between 0.7 and 2.7 mL/g/min^13^, ^22^, ^27^, ^51^ for global resting MBF. Those values are comparable to the obtained results using the bSSFP readout with uncorrected MBF calculation across all but one subject. Compared to the previously reported range for myoASL‐based MBF, uncorrected spGRE‐based MBF values were elevated (2.59 ± 1.37 mL/g/min). When calculated with the correction, however, spGRE‐based MBF (0.54–2.59 mL/g/min) was generally in line with this range and comparable to uncorrected bSSFP. The lowest observed perfusion values ranged at the lower end of MBF values reported in first‐pass perfusion literature (0.62–1.24 mL/g/min).^50^

With uncorrected calculation, spGRE‐based MBF showed higher PN, intra‐ and inter‐subject variability compared to bSSFP‐based MBF. This is in agreement with previous findings which demonstrate lower signal‐to‐noise ratio and temporal signal‐to‐noise ratio in cardiac imaging with spGRE snapshot imaging compared to bSSFP readout.^19^ Calculating spGRE‐based perfusion with the proposed correction tended to improve precision: Both intra‐subject variability and average PN from corrected spGRE readouts were on par with uncorrected bSSFP‐based values, while simultaneously providing reduced sensitivity to FA‐related effects. Similarly, the corrected spGRE approach resulted in less inter‐subject variability compared to both uncorrected bSSFP and spGRE.

Nonetheless, variability in those measurements remains high. This is likely due to PN, caused by temporal fluctuations of the blood flow. Changes in the heart rate can further induce timing variations within a control‐tag pair potentially impairing the variability if not accounted for. Lastly, residual motion after registration, such as caused by beat‐to‐beat variability or inconsistent breath‐holds, might add to the uncertainty in perfusion values. Further sequence development, such as free‐breathing or motion‐corrected acquisitions, and research into advanced postprocessing are warranted to address these sources of variability. With respect to diagnosis of myocardial ischemia, stress MBF cut‐off values ranged between 0.91 and 1.86 mL/g/min,^52^, ^53^ with stress MBF values in healthy volunteers of 1.97 up to 4.5 mL/g/min.^50^ Thus, an effect size of about 55% can be expected. The inter‐subject variability obtained in the present work, promises only moderate detection of those changes. Thus, further reduction of the variability in FAIR‐myoASL remains crucial for achieving diagnostic confidence as required in the clinic.

As it is common to ECG gated acquisitions, excessive heart rate variability in combination with inadequate gating windows can lead to imaging in different effective cardiac phases.^54^ Thus, in double ECG‐gated FAIR‐myoASL, this effect can lead to incongruence between the control and tag image. Due to the relatively stable duration of the systole compared to the diastole,^55^ however, recent studies suggest that systolic FAIR‐myoASL can offer higher robustness to such timing issues.^28^ Future studies in targeted cohorts, such as patients suffering from cardiac arrhythmia, are warranted to further investigate the suitability of systolic cardiac ASL in the clinic.

In the proposed work the correction was derived for the case of a FAIR‐ASL sequence. However, the proposed saturation‐baseline approach does not depend on the labeling mode and is applicable to other ASL schemes such as velocity^42^ or acceleration selective ASL.^56^ In fact, Zhang et al. proposed a similar approach to account for magnetization saturation in Look‐Locker FAIR‐myoASL (LL‐FAIR)^57^ using multivariate regression to eliminate the $T_{1}$ error. However, the performance was not compared to conventional fitting approaches and the proposed method was not explored in other myoASL sequences.

This study has several limitations. Current FAIR‐myoASL methods generally do not allow for extensive myocardial coverage since large inversion slabs can lead to increasing, nonnegligible transit delays.^43^, ^58^ Velocity selective labeling may allow for larger myocardial coverage as it is largely insensitive to transit delays, albeit with potential sensitivity to residual motion.^41^, ^42^ Future studies applying the proposed MBF calculation to velocity‐selective ASL are warranted. The FAIR‐myoASL sequence was acquired in healthy subjects at rest only and no stress perfusion has been obtained. Repeatability, as assessed by back‐to‐back scanning, presents only a subset of the factors influencing reproducibility or intra‐subject variability in a clinical setting. Further, the reproducibility and sensitivity of the corrected FAIR‐myoASL approach remain to be evaluated in patients with myocardial pathology. Due to the relatively small number of subjects included in this proof‐of‐principle study, larger studies assessing precision in a clinical setup or reproducibility over more extended time periods or different scan settings are warranted and would also allow for increased statistical power in comparing the uncorrected and corrected MBF calculation in bSSFP and spGRE readout. In this study, individual $T_{1,B}$ were obtained with MOLLI $T_{1}$‐mapping, which is known to underestimate $T_{1}$.^26^, ^59^ This could lead to inaccurate $T_{1,B}$ and, as shown in the results, impair the effectiveness of the proposed MBF calculation with individual $T_{1,B}$ to reduce the HR dependence of myoASL‐MBF. To that end, saturation based $T_{1}$ mapping sequences can be used in future work.^60^

## 6 CONCLUSION

Myocardial ASL can offer a contrast‐agent free alternative for myocardial perfusion assessment. Calculating myoASL‐MBF with inaccurate $T_{1,B}$ may lead to a mild heart rate dependence of MBF which was reduced by using individual $T_{1,B}$ values. Moreover, spurious MBF changes due to a varying acquisition flip angle were identified as the strongest confounder. With spGRE readouts, this effect was mitigated through the acquisition of an additional saturation‐baseline image. This approach can improve the robustness of myoASL and its potential clinical use in future.

## CONFLICT OF INTEREST STATEMENT

The authors declare no potential conflict of interests.

## Supporting information


**Figure S1.** Coefficient of variation of simulated myoASL‐MBF as a function of heart rate variability.
**Figure S2.** Simulated and phantom myoASL‐MBF as a function of the control‐tag delay.
**Figure S3.** Phantom myoASLMBF as a function of heart rate and blood *T*1.
**Figure S4.** Simulated myoASLMBF deviation as a function of acquisition flip angle and matrix size.
**Figure S5.** Simulated myoASLMBF as a function of blood *T*1 and *T*2 relaxation times.
**Figure S6.** Simulated myoASLMBF deviation as a function of blood *T*1 error.
**Figure S7.** Simulated myoASLMBF as a function of heart rate and blood *T*1.

## APPENDIX A SIGNAL EQUATIONS FOR BSSFP AND SPGRE READOUT

### A.1 bSSFP readout

The MMF for a bSSFP readout can be expressed via the transient state magnetization as described by Scheffler.^63^ For a readout with flip angle (FA) α and an $\frac{\alpha }{2}$‐preparation pulse, the magnetization vector after the k‐th RF pulse can be given as:^63^

(A1)
$$M(k)=(\sin(\frac{\alpha }{2})M_{0}-M_{\mathrm{ss}})\lambda_{1}^{k}+M_{\mathrm{ss}}\text{,}$$

with initial magnetization $M_{0}$, steady‐state magnetization $M_{ss}=\rho \frac{\sqrt{E_{2}}(1-E_{1})\sin(\alpha )}{1-(E_{1}-E_{2})\cos(\alpha )-E_{1}E_{2}}$, proton density ρ, coefficients $E_{1/2}=e^{-\text{TR}/T_{1/2}}$, and the eigenvalue $\lambda_{1}$:

(A2)
$$\begin{array}{ll} \lambda_{1} & =\cos(\alpha )(E_{1}-E_{2})+\sqrt{\cos^{2}(\alpha ){\left(E_{1}-E_{2}\right)}^{2}+4E_{1}E_{2}}\\ & \approx E_{1}\cos^{2}(\frac{\alpha }{2})+E_{2}\sin^{2}(\frac{\alpha }{2})\text{.} \end{array}$$

With $x=M_{z}(t_{0})$ the MMF becomes:

(A3)
$$f_{\text{MMF}}^{\text{bSSFP}}(x)=\sin(\frac{\alpha }{2})\lambda_{1}^{k}x+(1-\lambda_{1}^{k})M_{ss}.$$

### A.2 spGRE readout

Similarly, the MMF for a spGRE readout can be derived based on the Bloch equations. After a first RF pulse with FA α at $t_{0}$ and a second after a repetition time TR, the longitudinal magnetization $M_{z}(t_{0}+\text{TR})$ is given by

(A4)
$$M_{z}(t_{0}+\text{TR})=\cos^{2}(\alpha )E_{1}M_{z}(t_{0})+\cos(\alpha )(1-E_{1})M_{z,\text{eq}}\text{,}$$

with initial magnetization $M_{z}(t_{0})$ and equilibrium magnetization $M_{z,\text{eq}}$. After k RF pulses, the longitudinal magnetization yields

(A5)
$$\begin{array}{ll} M_{z}(k) & =\cos^{k}(\alpha )E_{1}^{-(k-1)}M_{z}(t_{0})+\\ & \;+\left(\sum_{i=0}^{k-2}{\left(\cos(\alpha )E_{1}\right)}^{-i}\right)\cos(\alpha )(1-E_{1}), \end{array}$$

and the MMF for spGRE can be given as

(A6)
$$\begin{array}{ll} f_{\text{MMF}}^{\text{spGRE}}(x) & =\cos^{k}(\alpha )E_{1}^{-(k-1)}x+\\ & \;+\frac{1-{\left(\cos(\alpha )E_{1}\right)}^{n-1}}{1-\cos(\alpha )E_{1}}(1-E_{1})\cos(\alpha )\rho M_{z,\text{eq}}\text{.} \end{array}$$

## APPENDIX B IMAGING SIGNAL FOR BASELINE, CONTROL AND TAG IMAGE IN FAIR‐MYOASL

In both, control and tag images, the myocardium is inverted in the imaging slice and recovers with $T_{1,M}$ during the time TI preceding the imaging readout. Using Equation (2), the myocardial signal contribution can thus be expressed as

(B1)
$$I_{M}=V_{M}(A_{M}x_{M}^{-}+B_{M})\text{,}$$

where $x_{M}^{-}=1-e^{-\text{TI}/T_{1,M}}$.

The blood signal, however, differs between the two settings. After the slice‐selective inversion in the control image, noninverted blood flows into the imaging slice with in‐flow rate $f_{\text{in}}$ giving rise to the signal contribution

(B2)
$$I_{C,B}^{+}=V_{B}f_{\text{in}}(A_{B}x_{B}^{+}+B_{B})\text{,}$$

with $x_{B}^{+}=1$. At the same time outflow of the initially inverted blood occurs with the same rate $f_{\text{in}}$, such that the remaining noninverted blood contributes to the signal with

(B3)
$$I_{C,B}^{-}=V_{B}(1-f_{\text{in}})(A_{B}x_{B}^{-}+B_{B})\text{,}$$

where $x_{B}^{-}=1-e^{-TI/T_{1,B}}$. Combining Equations (B2) and (B3) the full contribution of blood signal to the control image yields:

(B4)
$$I_{C,B}=V_{B}(f_{\text{in}}(A_{B}x_{B}^{+}+B_{B})+(1-f_{\text{in}})(A_{B}x_{B}^{-}+B_{B}))\text{.}$$

In tag images, the in‐flowing blood is also inverted due to the non‐selective inversion, and can be expressed as

(B5)
$$I_{T,B}=V_{B}(A_{B}x_{B}^{-}+B_{B})\text{.}$$

Finally, in the case of the baseline image, neither myocardium nor blood is inverted ($x_{M}^{+}=x_{B}^{+}=1$):

(B6)
$$\begin{array}{ll} I_{\text{BL},B} & =V_{B}(A_{B}x_{B}^{+}+B_{B})\\ I_{M,\text{BL}} & =V_{M}(A_{M}x_{M}^{+}+B_{M}). \end{array}$$

Combining the signal contributions from Equations (B1)–(B6), yields the following signal equations for the control ($I_{C}$), tag ($I_{T}$), and baseline signal ($I_{\text{BL}}$) in FAIR‐myoASL:

(B7)
$$\begin{array}{ll} I_{C} & =I_{M}+I_{C,B}=\\ & =V_{M}(A_{M}x_{M}^{-}+B_{M})\\ & \;+V_{B}(f_{\text{in}}(A_{B}x_{B}^{+}+B_{B})+(1-f_{\text{in}})(A_{B}x_{B}^{-}+B_{B}))\text{,} \end{array}$$

(B8)
$$\begin{array}{ll} I_{T} & =I_{M}+I_{T,B}=\\ & =V_{M}(A_{M}x_{M}^{-}+B_{M})+V_{B}(A_{B}x_{B}^{-}+B_{B})\text{,} \end{array}$$

(B9)
$$\begin{array}{ll} I_{\text{BL}} & =I_{M,\text{BL}}+I_{\text{BL},B}=\\ & =V_{M}(A_{M}x_{M}^{+}+B_{M})+V_{B}(A_{B}x_{B}^{+}+B_{B})\text{.} \end{array}$$

Subtracting this saturation‐baseline from the original baseline image yields

(B10)
$$\begin{array}{ll} I_{\text{BL}}-I_{\mathrm{BL},\mathrm{Sat}} & =V_{M}(A_{M}x_{M}^{+}+B_{M})+V_{B}(A_{B}x_{B}^{+}+B_{B})\\ & \;-(V_{M}(A_{M}\cdot 0+B_{M})+V_{B}(A_{B}\cdot 0+B_{B}))=\\ & =V_{M}A_{M}x_{M}^{+}+V_{B}A_{B}x_{B}^{+}\text{.} \end{array}$$
