# Supplementary material for: Improved reproducibility for myocardial ASL: Impact of physiological and acquisition parameters
Source: Magn Reson Med. 2023 Sep 5;91(1):118–32. doi: 10.1002/mrm.29834 (PMC10962577; doi:10.1002/mrm.29834)
Supplement: Supplementary file 1 — Figure S1. Coefficient of variation of simulated myoASL‐MBF as a function of heart rate variability. Figure S2. Simulated and phantom myoASL‐MBF as a function of the control‐tag delay. Figure S3. Phantom myoASLMBF as a function of heart rate and blood T1. Figure S4. Simulated myoASLMBF deviation as a function of acquisition flip angle and matrix size. Figure S5. Simulated myoASLMBF as a function of blood T1 and T2 relaxation times. Figure S6. Simulated myoASLMBF deviation as a function of blood T1 error. Figure S7. Simulated myoASLMBF as a function of heart rate and blood T1. [file MRM-91-118-s001.pdf]

SUPPLEMENTARY MATERIAL FOR:

Improved reproducibility for myocardial ASL: Impact of physiological and acquisition parameters

Maša Božić-Iven[1, 2], Stanislas Rapacchi[3], Qian Tao[2], Iain Pierce[4], George Thornton[4,5], Christian Nitsche[4,5,6], Thomas Treibel[4,5], Lothar R. Schad[1], and Sebastian Weingärtner[2]

[1]Computer Assisted Clinical Medicine, Medical Faculty Mannheim, Heidelberg University, Mannheim, Germany
[2]Magnetic Resonance Systems Lab, Department of Imaging Physics, Delft University of Technology, Delft, Netherlands
[3]Centre de Resonance Magnetique Biologique et Medicale, CNRS, Aix Marseille Université, Marseille, France
[4]Barts Heart Centre, Barts Health NHS Trust, London, United Kingdom
[5]Institute of Cardiovascular Science, University College London, London, United Kingdom
[6]Division of Cardiology, Medical University of Vienna, Vienna, Austria

INDEX OF SUPPLEMENTARY MATERIALS

Numerical Simulations: Methods 2

Numerical Simulations: Results 2

HR Variability and MBF Quantification 3

INDEX OF SUPPLEMENTARY FIGURES

Figure S1 Coefficient of variation of simulated myoASL-MBF as a function of heart rate variability. 3

Figure S2 Simulated and phantom myoASL-MBF deviation as a function of the control-tag delay. 4

Figure S3 Phantom myoASL-MBF as a function of heart rate and blood T_1. 5

Figure S4 Simulated myoASL-MBF as a function of acquisition flip angle and matrix size. . . 6

Figure S5 Simulated myoASL-MBF as a function of blood T_1 and T_2 relaxation times. 7

Figure S6 Simulated myoASL-MBF deviation as a function of blood T_1 error. 8

Figure S7 Simulated myoASL-MBF as a function of heart rate and blood T_1. 9

Numerical Simulations: Methods

To assess the effect of physiological and acquisition related parameters on myoASL-based MBF values, the FAIR-myoASL sequence was numerically simulated using Bloch equation simulations. All numerical simulations were performed in MATLAB. Both, bSSFP and spGRE readouts were simulated. If not indicated otherwise, general sequence parameters in simulation were: ramp-up pulses 10/0 (bSSFP/spGRE), FA 50°/35° (bSSFP/spGRE), 100 readout pulses (n = 50), 100 % inversion efficiency, 6 s control-tag delay. A blood volume fraction of 0.14 [1] and a blood replacement/in-flow rate of 0.29 1/s were simulated, resulting in an effective MBF input value of 2.4 ml/g/min. The assumed in-flow rate corresponds to about 4 ml/s for a myocardial blood volume of 15 ml (about 10 % of the left-ventricular mass [2], [3]). Other physiological parameters were simulated as: HR 60 bpm, blood T_1/T_2 relaxation times at 3 T of 2000 ms/250 ms, and myocardial T_1/T_2 relaxation times of 1460 ms/45 ms [4], [5], unless otherwise specified.

To simulate the effect of in-flow during TI and to obtain the control and tag signal, the inverted and native signal of blood and myocardium were combined according to Eqs. B13 and B14. The conventional and saturation-baseline signal (in spGRE) were calculated using Eq. B15.

As in the phantom experiments, three simulation experiments were performed to investigate the effect of physiological and acquisition parameters, respectively. For the first set of simulated myoASL-MBF data, a FA range of 1° - 80° in bSSFP and 1° - 40° in spGRE was simulated for AMSs between 100 and 220. For the second data set, the HR was varied between 40 and 120 bpm (RR duration 500 - 1500 ms) and different combinations of representative T_1/T_2 relaxation times corresponding to those of the selected phantom vials were used. For the third simulation experiment, the control-tag delay was varied between 6 and 12 s with fixed physiological and sequence parameters as described above.

Numerical Simulations: Results

Flip Angle

MBF was simulated for a range of acquisition FAs and AMSs in bSSFP and spGRE readout as depicted in Supporting Information Figure S4. For bSSFP readouts with uncorrected MBF calculation, there was a strong correlation with FA across all simulated T_1 values ($R^2 = 1$). Simulated MBF was overestimated with increasing FA and with increasing AMS (slope: 0.023-0.056) except for T_1/T_2 of 1770/45 ms where MBF was underestimated with FA and AMS (slope: -0.004 (AMS 120)/-0.021 (AMS 256)). The same FA dependence was observed in bSSFP-based MBF with $T_{1,B}$ corrected calculation ($R^2 = 1$). For the case of uncorrected spGRE, MBF was largely constant up to FAs of about 5°. With increasing FA beyond 5°, MBF was increasingly underestimated and reached zero for FAs of about 28° and larger with an AMS of 120, and about 18° with an AMS of 256 ($R^2 = 1$). With fully corrected calculation, spGRE-based MBF was largely constant over the entire range of FAs (2.75/2.58 ml/g/min AMS 120/AMS 256). The correlation with FA was reduced to $0.0 < R^2 < 0.30$ with AMS 120 and $0.11 < R^2 < 0.44$ with AMS 256.

Blood T_1 and T_2 Relaxation Time

Simulated MBFs as a function of blood T_1 ($T_{1,B}$) and T_2 relaxation time for the four combinations of readout and calculation are shown in Supporting Information Figure S5. While spGRE-based MBF is constant over the range of simulated blood T_2 values, bSSFP-based MBF showed a strong non-linear relation. MBF obtained with bSSFP and spGRE readouts showed a moderate dependence on $T_{1,B}$ increasing by about 0.16/0.08 ml/g/min per 100 ms in $T_{1,B}$, which is eliminated when using the correct $T_{1,B}$ in calculation. Increasing measurement errors in $T_{1,B}$ led to increasing MBF errors (approximately 3% per 100ms) for all four readout and calculation combinations (Supporting Information Figure S6). Further, if an inaccurate $T_{1,B}$ is used in calculation, MBF shows a weak HR dependence in both readouts (on average 0.01 ml/g/min per 100 ms change in RR) as illustrated in Supporting Information Figure S7. This is alleviated when MBF is calculated with true $T_{1,B}$.

HR variability and MBF quantification

The quantification of the MBF is based on Buxton's GKM and usually adapted to a double ECG-triggered sequence [6], [7]:

$$MBF = \frac{\lambda}{\delta \cdot I_{BL}} \left(\frac{I_C}{TI_C \cdot e^{-TI_C/T_{1,B}}} - \frac{I_T}{TI_T \cdot e^{-TI_T/T_{1,B}}} \right) \tag{1}$$

with control, tag, baseline signal I_C, I_T, I_{BL}, and the corresponding inversion times TI_C and TI_T, inversion efficiency $\delta = 1 - \cos(\alpha_{inv})$, blood-water partition coefficient $\lambda = 1ml/g$ [8], [9] and blood T_1 relaxation time $T_{1,B}$. In a simulation experiment with six control/tag pairs and 1000 repetitions, we evaluated the MBF quantification with individual TI_C/TI_T, an average inversion time $\overline{TI}$ for each control/tag pair, and a global average $\overline{TI}$ per sequence. As shown in Figure S1, the MBF variation is comparable in the face of heart rate (HR) variations when the true $TI_{C/T}$ or an averaged TI is used. Therefore, we can further resort to the original model for a single-gated myoASL sequence

$$MBF = \frac{\lambda \cdot (I_C - I_T)}{\delta \cdot I_{BL} \cdot \overline{TI} \cdot e^{-\overline{TI}/T_{1,B}}} \ . \tag{2}$$

with a pairwise averaged inversion time $\overline{TI}$ for each control/tag image pair.

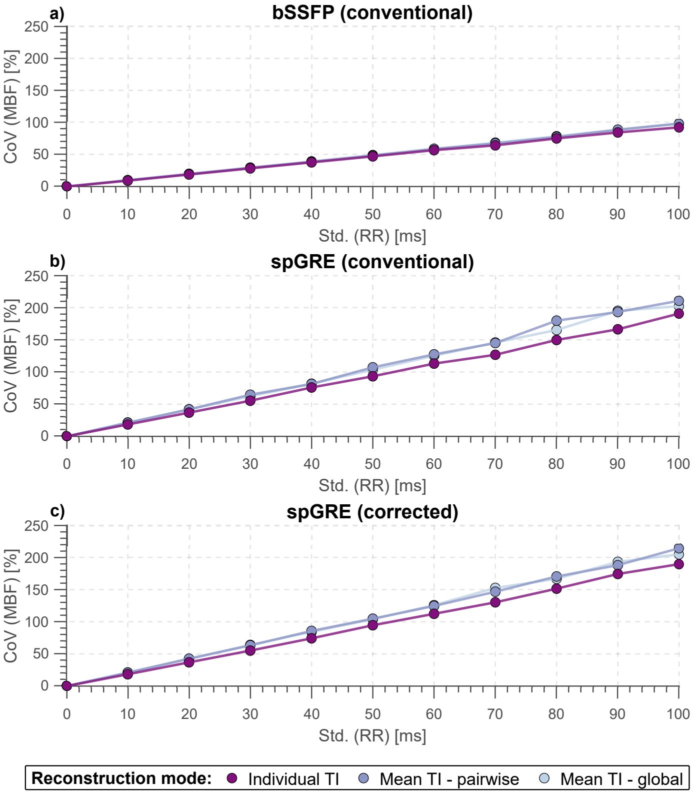

Figure S1: Coefficient of variation of simulated myoASL-MBF from **a)** uncorrected bSSFP and **b)** spGRE readouts as well as corrected spGRE readouts as a function of simulated heart rate variability. Calculating bSSFP-based MBF with a pairwise inversion time ($\overline{TI}$) for each control/tag pair leads to negligible differences compared to using individual TIs. For spGRE readout, MBF deviations increase by 10% when pairwise or globally averaged $\overline{TI}$ instead of individual TIs are used in MBF quantification. Moreover, the proposed correction does not lead to a substantial increase in MBF deviations compared to the uncorrected case.

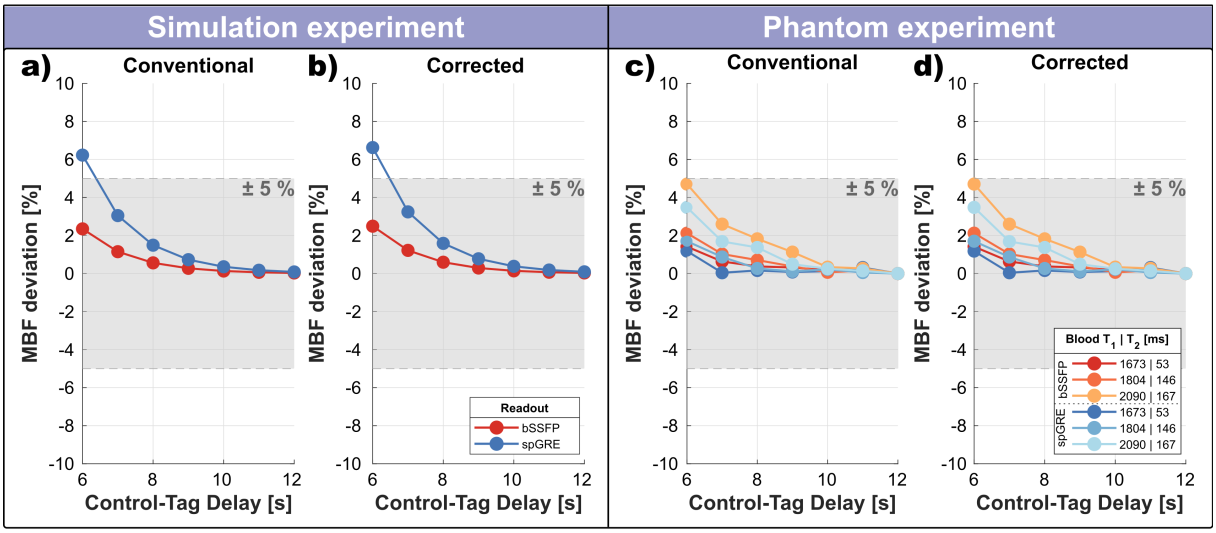

Figure S2: Relative MBF deviation from the asymptotic MBF (very long control-tag delay "∞") as a function of the control-tag delay for bSSFP and spGRE readout, from **a), b)** simulation and **c), d)** phantom experiments. MBF was calculated with **a), c)** fixed and **b), d)** individual blood T_1 ($T_{1,B}$). Additionally, for corrected spGRE (**d)**), the saturation-baseline approach as proposed in this work was used in MBF calculation. Simulated MBF deviation decreases with increasing delay and reaches 0% at about 10 s (simulated $T_{1,B}$: 1900 ms). In phantom, MBF deviation reaches 0% from delays of about 9 s for all readout/quantification combinations and for all phantom vials (i.e. $T_{1,B}$ values). In phantom, the difference between a 6 s long delay and the steady state was $< 4\%$ in bSSFP and $< 5\%$ in spGRE readouts, such that a control-tag delay of 6 s was chosen.

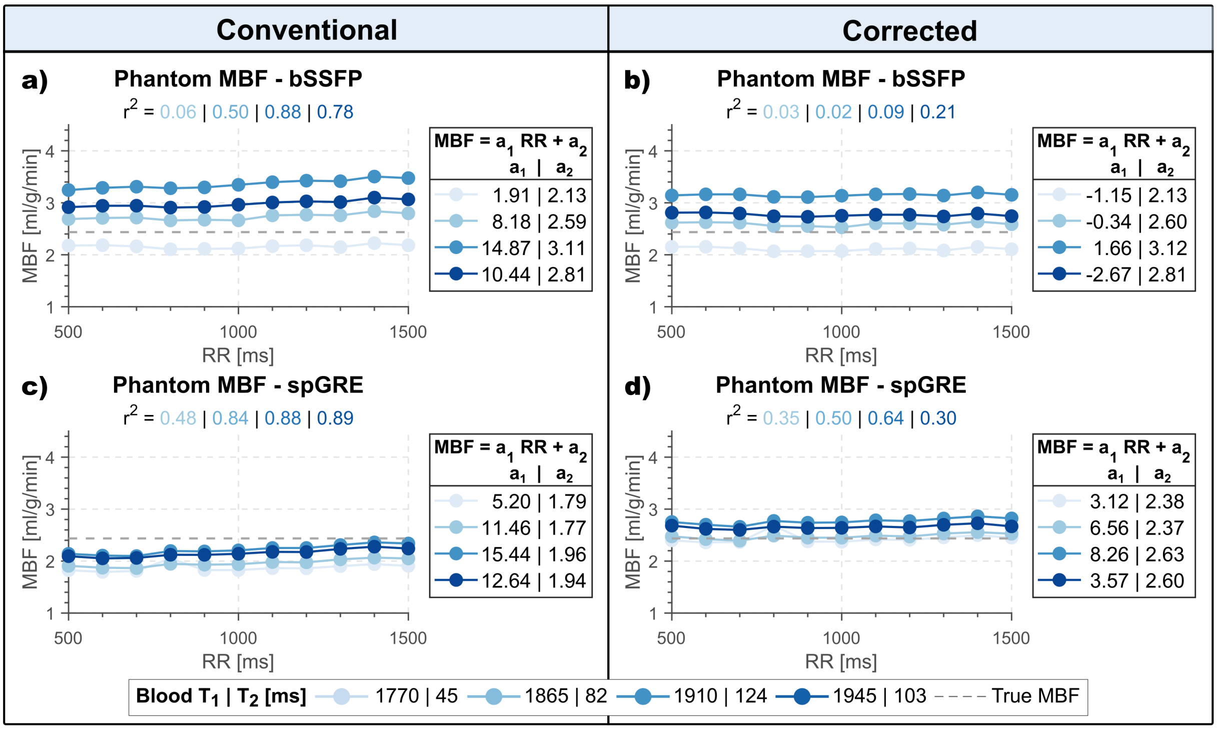

Figure S3: Phantom myoASL-MBF from **a), b)** bSSFP and **c), d)** spGRE readout. MBF was calculated with **a), c)** fixed and **b), d)** individual blood T_1 ($T_{1,B}$). Additionally, for corrected spGRE (**d)**), the saturation-baseline approach as proposed in this work was used in MBF calculation. MBF is plotted against the simulated heart rate (HR) for four phantom vials corresponding to different blood T_1 ($T_{1,B}$) and T_2 relaxation times. The slope (a_1) and intercept (a_2) for each vial are obtained from linear regression. Phantom MBF from both readouts shows a HR dependence if the $T_{1,B}$ used in quantification differs from the true (vial) $T_{1,B}$. The HR dependence is significantly reduced when MBF is calculated with true $T_{1,B}$ as proposed in this work. Differences between the different vials remains for corrected bSSFP due to the differences in T_2 relaxation times.

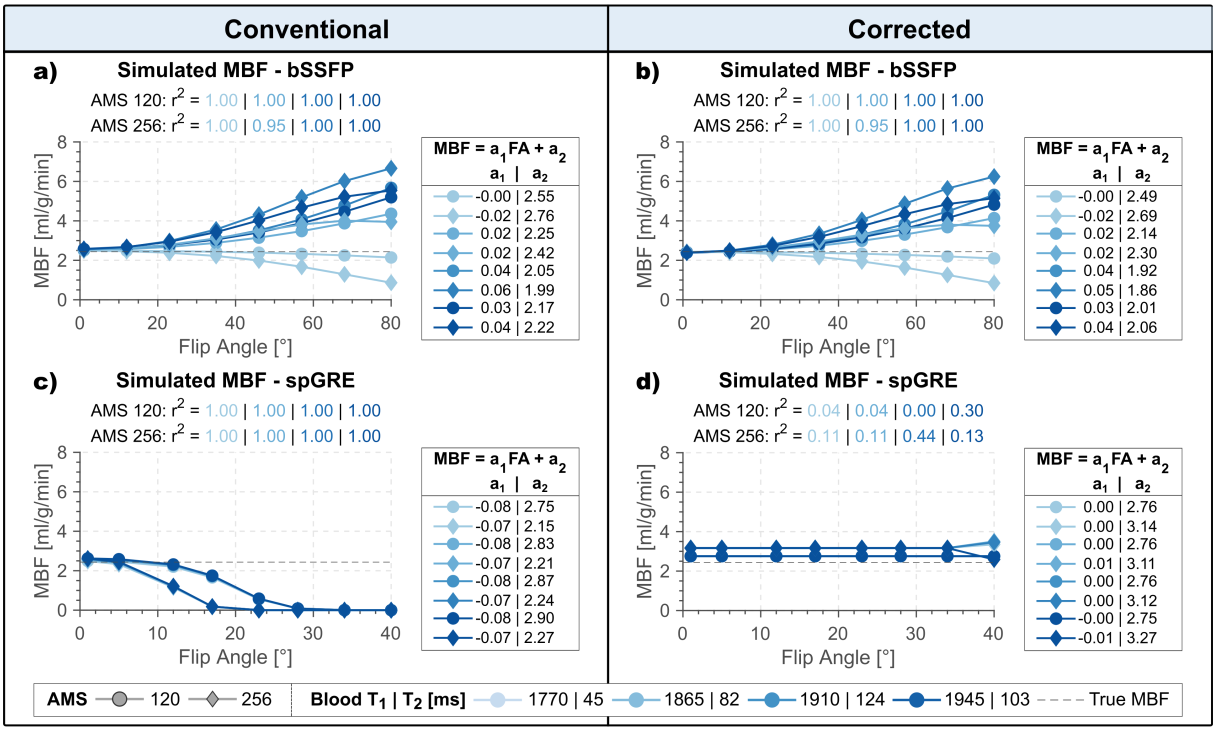

Figure S4: Simulated myoASL-MBF from **a), b)** bSSFP and **c), d)** spGRE readout. MBF was calculated with **a), c)** fixed and **b), d)** individual blood T_1 ($T_{1,B}$). Additionally, for corrected spGRE (**d)**), the saturation-baseline approach as proposed in this work was used in MBF calculation. MBF is shown as a function of acquisition flip angle (FA) for two acquisition matrix sizes (AMS). The slope (a_1) and intercept (a_2) are obtained from linear regression. A strong dependence of bSSFP- and spGRE-based MBF on FA is observed which is enhanced for larger AMS. Using the proposed correction eliminates the FA dependence for spGRE readouts.

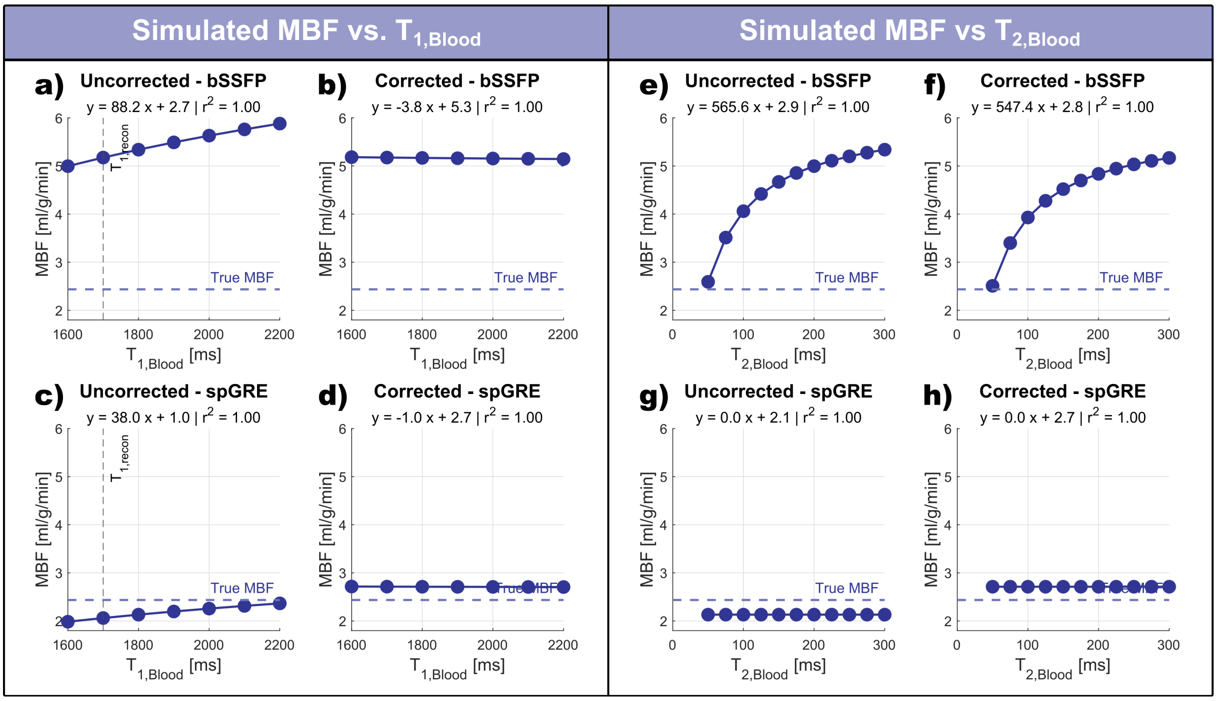

Figure S5: Simulated myoASL-MBF as a function of blood **a)-d)** T_1 and **e)-g)** T_2 relaxation times from **a), b), e), f)** bSSFP and **c), d) g), h)** spGRE readouts. MBF was calculated with **a), c), e), g)** fixed and **b), d), f), h)** individual blood T_1 ($T_{1,B}$). Additionally, for corrected spGRE (**d), h)**), the saturation-baseline approach as proposed in this work was used in MBF calculation. Simulated MBF increased with increasing blood T_1 for uncorrected bSSFP and spGRE readouts. When the proposed correction was used, MBF from both readouts decreased slightly with increasing T_1 due to the effect of the long acquisition matrix size in snapshot readout. For both quantification methods, bSSFP-based MBF strongly increased with increasing blood T_2, while spGRE-based MBF was constant over the range of simulated T_2 values.

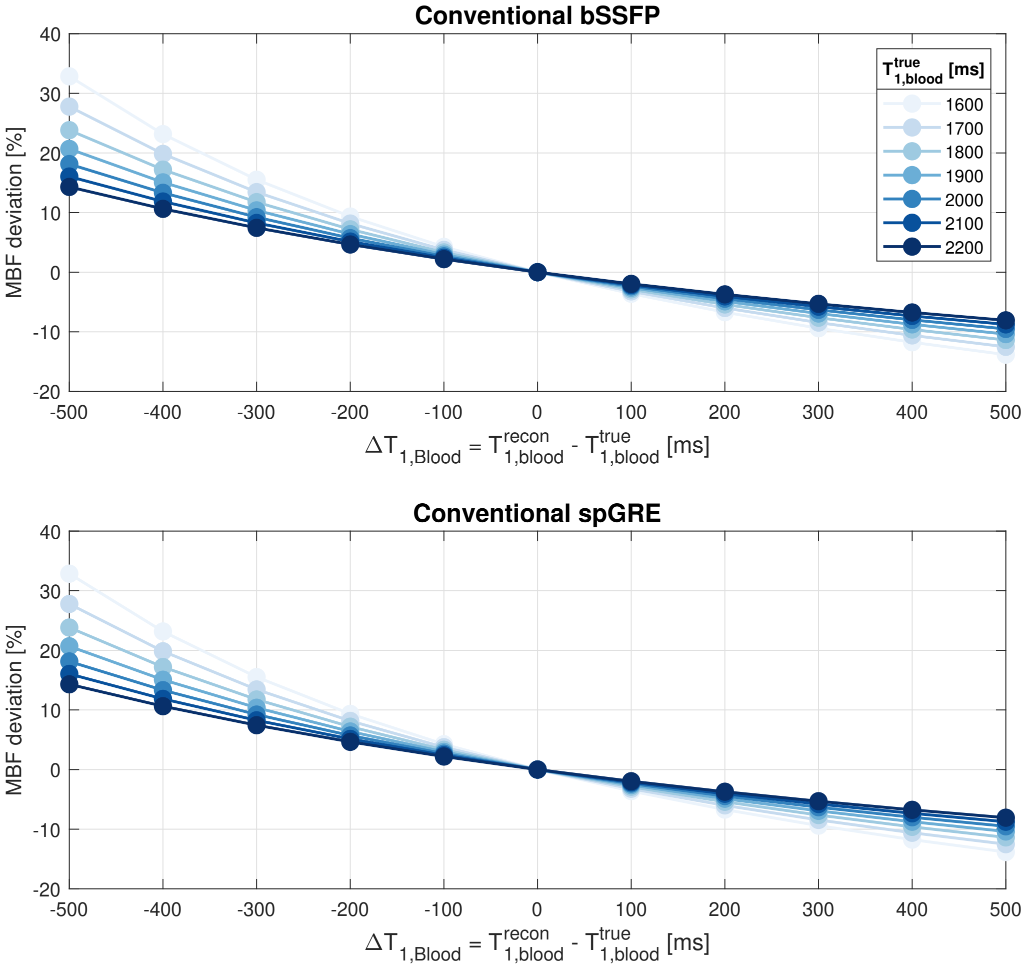

Figure S6: Simulated myoASL-MBF deviation as a function of the measurement error of blood T_1 ($T_{1,B}$) for a range of $T_{1,B}$ values. If $T_{1,B}$ is quantified incorrectly, an error of approximately 3% per 100 ms of blood $T_{1,B}$ deviation in MBF is accrued with conventional (uncorrected) MBF calculation for both bSSFP (top) and spGRE (bottom) readouts.

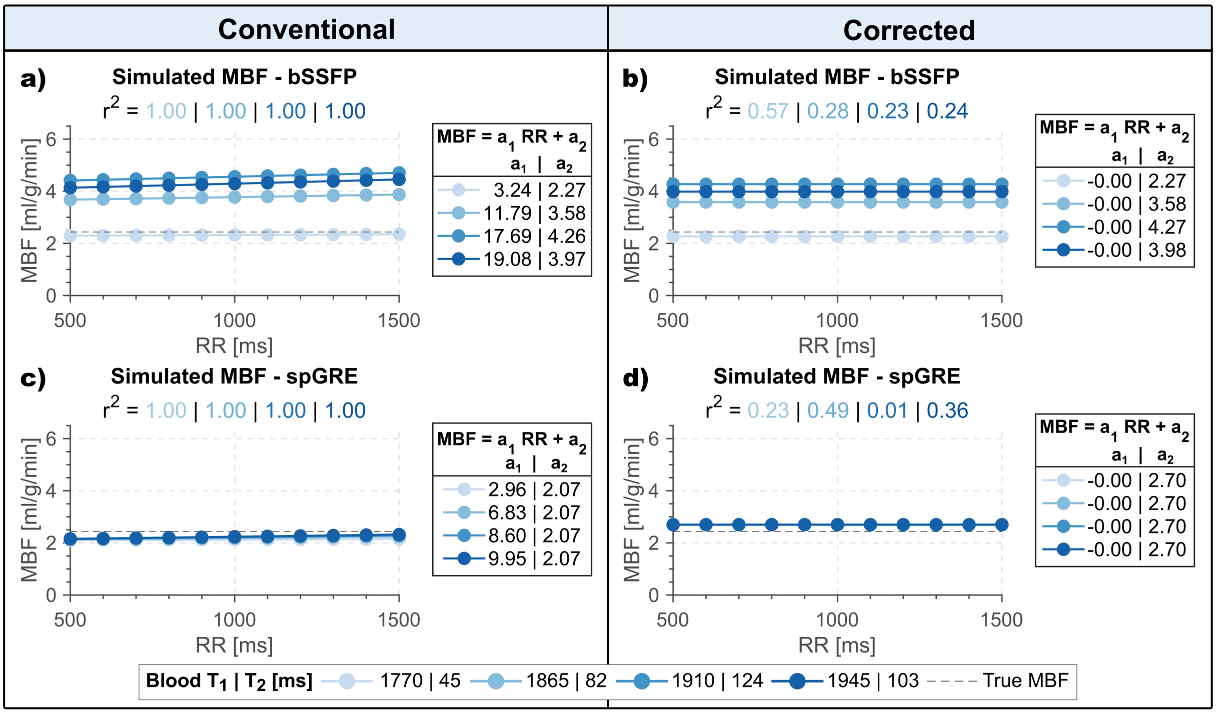

Figure S7: Simulated myoASL-MBF from **a), b)** bSSFP and **c), d)** spGRE readout. MBF was reconstructed with **a), c)** fixed and **b), d)** individual blood T_1 ($T_{1,B}$). Additionally, for corrected spGRE (**d)**), the saturation-baseline approach as proposed in this work was used in reconstruction. MBF is shown as a function of heart rate (HR) for different blood T_1 ($T_{1,B}$) and T_2 relaxation times. The slope (a_1) and intercept (a_2) are obtained from linear regression. If an inaccurate $T_{1,B}$ is used in reconstruction, MBF shows a HR dependence in both readouts. This is alleviated when MBF is reconstructed with true $T_{1,B}$ as proposed in this work.

References

[1] C. M. Wacker, F. Wiesmann, M. Bock, et al., “Determination of regional blood volume and intra-extracapillary water exchange in human myocardium using feruglose: First clinical results in patients with coronary artery disease,” Magnetic Resonance in Medicine, vol. 47, no. 5, pp. 1013–1016, 2002.

[2] J. Nickander, R. Themudo, S. Thalén, et al., “The relative contributions of myocardial perfusion, blood volume and extracellular volume to native t1 and native t2 at rest and during adenosine stress in normal physiology,” Journal of Cardiovascular Magnetic Resonance, vol. 21, no. 1, pp. 1–10, 2019.

[3] K. Mizukoshi, M. Takeuchi, Y. Nagata, et al., “Normal values of left ventricular mass index assessed by transthoracic three-dimensional echocardiography,” Journal of the American Society of Echocardiography, vol. 29, no. 1, pp. 51–61, 2016.

[4] I. Hermann, P. Kellman, O. B. Demirel, M. Akçakaya, L. R. Schad, and S. Weingärtner, “Free-breathing simultaneous t1, t2, and t2* quantification in the myocardium,” Magnetic Resonance in Medicine, vol. 86, no. 3, pp. 1226–1240, 2021.

[5] S. Weingärtner, N. M. Meßner, J. Budjan, et al., “Myocardial t1-mapping at 3t using saturation-recovery: Reference values, precision and comparison with molli,” Journal of Cardiovascular Magnetic Resonance, vol. 18, no. 1, pp. 1–9, 2017.

[6] R. B. Buxton, L. R. Frank, E. C. Wong, B. Siewert, S. Warach, and R. R. Edelman, “A general kinetic model for quantitative perfusion imaging with arterial spin labeling,” Magnetic Resonance in Medicine, vol. 40, no. 3, pp. 383–396, 1998, ISSN: 07403194. DOI: 10.1002/mrm.1910400308.

[7] H. P. Do, A. J. Yoon, M. W. Fong, F. Saremi, M. L. Barr, and K. S. Nayak, “Double-gated myocardial ASL perfusion imaging is robust to heart rate variation,” Magnetic Resonance in Medicine, vol. 77, no. 5, pp. 1975–1980, 2017, ISSN: 15222594. DOI: 10.1002/mrm.26282.

[8] K. S. McCommis, T. A. Goldstein, H. Zhang, B. Misselwitz, R. J. Gropler, and J. Zheng, “Quantification of myocardial blood volume during dipyridamole and doubtamine stress: A perfusion cmr study,” Journal of Cardiovascular Magnetic Resonance, vol. 9, no. 5, pp. 785–792, 2007.

[9] S. Bergmann, K. Fox, A. Rand, et al., “Quantification of regional myocardial blood flow in vivo with h215o.,” Circulation, vol. 70, no. 4, pp. 724–733, 1984.
